# Anticipatory postural adjustments in older versus young adults: a systematic review and meta-analysis

**DOI:** 10.1186/s13643-022-02116-x

**Published:** 2022-11-23

**Authors:** Manuela Brito Duarte, Gizele Cristina da Silva Almeida, Kelly Helorany Alves Costa, Daniela Rosa Garcez, Anselmo de Athayde Costa e Silva, Givago da Silva Souza, João Simão de Melo-Neto, Bianca Callegari

**Affiliations:** 1 Laboratory of Human Motricity Sciences, Federal University of Pará, Av. Generalíssimo Deodoro 01, Belém, Pará 66050-160 Brazil; 2 Master’s Program in Human Movement Sciences, Federal University of Pará, 448/475 Av. Generalíssimo Deodoro 01, Belém, Pará 66050-160 Brazil; 3 Tropical Medicine Center, Federal University of Pará, Av. Generalíssimo Deodoro 92, Belém, Pará 66050-240 Brazil; 4 University Hospital Bettina Ferro de Souza, Federal University of Pará, Rua Augusto Corrêa, n 1. Cep 66075-110, Guamá, Belém, Pará Brazil; 5 Neuroscience and Cell Biology Graduate Program (PPGNBC), Federal University of Pará, Rua Augusto Corrêa, n 1. Cep 66075-110, Guamá, Belém, Pará Brazil

**Keywords:** Frailty, Older adults, Stability, Posture, Surface electromyography, Meta-analysis

## Abstract

**Background:**

Anticipatory postural adjustments (APAs) are a feedforward mechanism triggered in advance to a predictable perturbation, to help the individual counteract mechanical effects that the disturbance may cause. Whether or not this strategy is compromised in the elderly is not a consensus in the literature.

**Methods:**

In this systematic review with meta-analysis, we investigated aging effects on postural control, based on anticipatory postural adjustments (APAs). We selected 11 eligible articles of the following databases: Lilacs, SciELO, PubMed, Cochrane Central, Embase, and CINAHL, involving 324 research participants, assessing their methodological quality and extracting electromyographic, posturographic, and kinematic measurements. We included studies that investigated the occurrence of APAs in healthy younger and older adults, published before 10th August 2022, in English. Studies involving participant with conditions that may affect balance or that did not report measures of onset or amplitude of electromyography (EMG), COP, or kinematics were excluded. To analyze the aggregated results from these studies, we performed the analysis based on the outcome measures (EMG, COP, or kinematic measures) used in individual studies. We calculated differences between younger and older adult groups as the mean differences between the groups and the estimated effect. Egger’s test was conducted to evaluate whether this meta-analysis had publication bias.

**Results:**

Through this review, older adults showed no significant difference in the velocity to perform a movement compared to the younger adults (*M*D 0.95, 95% *CI* −0.86, 2.76, *I*^2^ = 82%), but both muscle onset and center of pressure (COP) onset were significantly more delayed in older than in younger adults: erector spinae (*MD* −31.44, 95% *CI* −61.79, −1.09, *I*^2^ = 95%); rectus abdominis (RA) (*MD* −31.51, 95% *CI* −70.58, −3.57, *I*^2^ = 85%); tibialis anterior (TA) (*MD* −44.70, 95% *CI* −94.30, 4.91, *I*^2^ = 63%); soleus (SOL) (*MD* −37.74, 95% *CI* −65.43, −10.05, *I*^2^ = 91%); gastrocnemius (GAS) (*MD* −120.59, 95% *CI* −206.70, −34.49, *I*^2^ = 94%); quadriceps (Q) (*MD* −17.42, 95% *CI* −34.73, −0.12, *I*^2^ = 0%); biceps femoris (BF) (*MD* −117.47, 95% *CI* −192.55, −42.70, *I*^2^ = 97%); COP onset (*MD* −45.28, 95% *CI* −89.57, −0.98, *I*^2^ = 93%), and COP apa (COPapa) (*MD* 2.35, 95% *CI* −0.09, 4.79, *I*^2^ = 64%). These changes did not seem to be linked to the speed of movement but possibly to age-related physiological changes that indicated decreased motor control during APAs in older adults.

**Conclusions:**

Older adults use different postural strategies that aim to increase the safety margin and stabilize the body to perform the movement, according to the requirements imposed, and this should be considered in rehabilitation protocols.

**Systematic review registration:**

PROSPERO CRD420119143198

**Supplementary Information:**

The online version contains supplementary material available at 10.1186/s13643-022-02116-x.

## Introduction

Aging is defined as a continuous and irreversible process associated with changes that lead to reduced functional capacity and levels of physical activity [1]. These processes include structural and motor changes in reflexes, proprioception, balance, postural, and motor control [2, 3] that, when added to social and environmental factors, contribute to an emergence of instability and increased fall risk [4]. Falls are an important public health problem in the older adults, due to their frequency and high socioeconomic cost, especially when they result in loss of independence and institutionalization [5].

Balance involves maintaining the body’s center of mass over its base of support while executing motor actions, typically in a bipedal stance, and it is fundamental for daily living activities [6]. Postural control strategies are divided into pre- and post-disturbance moments, known as “predictive” (anticipatory) and “reactive” (compensatory) postural control, respectively [7]. When a threat to balance (a perturbation) is predicted, a feedforward mechanism is triggered in advance, to help the individual counteract mechanical effects that the disturbance may cause. In this mechanism, the postural muscles are recruited before the disturbance, in a strategy called anticipatory postural adjustments (APAs) [8–10]. Past research has shown that APAs may be compromised in older adults [11–15] due to a decline in physiological phenomena in several systems that is characteristic of the senescence process and a physiological explanations for increased fall risk in this population [16].

Some studies using different motor tasks to generate APAs in older adults have suggested that these APAs are significantly smaller in amplitude and delayed, when compared to young people. In these studies, postural muscle recruitment started very close to the moment of disturbance and with limited magnitude, which indicated a failure in the advance planning of motor control among older adults [11, 13, 15–18]. However, there are disagreements among researchers on this point, and other studies found no difference between younger and older groups in the electromyographic parameters of the postural muscles [14, 19].

The pattern by which postural muscles are recruited, usually described as from distal to proximal in young people [13], may be at least slightly different among older adults. Using a task of pulling and pushing a rod, Inglin and Woollacott [11] found a pattern of distal to proximal muscle recruitment in both older and younger adults, but older participants jointly activated the dorsal and the ventral muscles of the lower limbs, while younger participants primarily activated the ventral muscles. In addition, the center of pressure (COP), measured by force platform, has also been a source of controversy when comparing older and younger adults through APA investigations. While young people seemed to initiate a displacement of the COP before the moment of disturbance, older adults showed a more reactive than anticipatory response, increasing their destabilization [11, 12, 18, 20]. Other studies found no significant differences in COP onset related to aging [15, 19, 21].

Despite these variant results, few systematic reviews of APA research have scrutinized the effects of aging on the APAs of motor planning and, thus, have focused on the relationship between APAs and sitting [8] or addressed specific diseases, such as multiple sclerosis [22], stroke [23], or patients with low back pain [24]. To fill this void, we intended this review to summarize and analyze aggregated data from prior research to determine whether APAs are altered in healthy older adults, compared to young people, using muscle activity, center of pressure (COP), and kinematics features as variables of particular interest.

## Method

We prepared this systematic review of the APA literature in the methodological context of the declaration of Preferred Reported Items for Systematic Review and Meta-Analysis (PRISMA) guidelines, according checklist presented in Supplementary Table S1. We registered this review with the international prospective register of systematic reviews (PROSPERO) under the code CRD420119143198 (to be removed in the blinded copy).

### Information sources and search strategy

We first carried out a bibliographic survey of the following databases: Lilacs, SciELO, PubMed, Cochrane Central, Embase, Scopus, and Web of Science. Unpublished manuscripts and conference abstracts were also not eligible. Available data, published before 10th August 2022, in English was searched following previously defined criteria according to the PICOS acronym (population, intervention, comparison, outcome, and study type). We considered articles describing APAs in both healthy and older adults using the following descriptors: anticipatory postural adjustment (s), APA (s), age(d), older, aging, elderly, and older adults. Search strategy was performed as depict in Supplementary Table S2.

### Study eligibility criteria

We included observational studies of cross-sectional type that investigated the occurrence of APAs in healthy younger and older adults. Studies are eligible for inclusion if written in English and were classified as II-IV level of evidence on the National Health and Medical Research Council (NHMRC) hierarchy of evidence for international studies [25]. We required selected studies (a) to have investigated APAs in younger adults (18 to 40 years old) and older adults (over 60 years old), (b) to have verified the onset and amplitude of muscle recruitment (electromyography), and (c) to have included COP parameters (force platform) or kinematics features during the period of APAs. We excluded studies with exacerbated postural reaction after the disturbance (such as falls and the use of the step to return to initial position). Studies involving animals, pregnant women, and participants with any diagnoses that affected balance such as a neurological or orthopedic disease and metabolic or inflammatory disorders were also excluded. Table 1 summarizes the inclusion and exclusion criteria according to the PICOS acronym (population, intervention, comparison, outcome, and study type).

**Table 1:** Summary of inclusion and exclusion criteria

Study characteristic	Inclusion criteria	Exclusion criteria
Study design	Observational, cross-sectional studies	Non-English language publication, published before 10thAugust 2022
Population	Younger adults (18 to 40 years old) and older adults (over 60 years old)	Animals, pregnant women, and participants with any diagnoses that affected balance such as a neurological or orthopedic disease, and metabolic or inflammatory disorders, were also excluded
Interventions	Anteroposterior perturbation (including unilateral arm movements, pendulum impact, tilt in force platform)	Gait initiation and any protocol wit exacerbated response
Comparators	Younger vs older adults	Pathological conditions
Setting	APAs assessments including onset, amplitude of muscle recruitment (electromyography) and/or COP parameters (force platform), and/or kinematics features	Exacerbated postural reaction after the disturbance (such as falls and the use of the step to return to initial position)
Outcome	APA onset and/or APA amplitude (any muscle) COP onset and/or COP amplitude	None

*APAs*, anticipatory postural adjustments; *COP*, center of pressure

### Selection process

Two independent reviewers examined the studies to determine their relevance, firstly by title and abstract and, when necessary, by the full text. Next, reviewers read the full text of each article and applied the abovementioned inclusion and exclusion criteria. In cases where the reviewers did not agree with an article’s inclusion, a third reviewer acted as an arbitrator.

### Methodological quality of articles

To avoid the risk of bias in the included studies, we determined degree of agreement among reviewers for assessing methodological quality by using the McMaster Critical Review form for Quantitative Studies [26]. Reviewers were instructed to answer a questionnaire with 16 items according to the recommended guidelines. The maximum possible score was 16, indicating excellent methodological quality. Article scores were then divided into five categories, dependent on the score received: poor (score ≤ 8), fair (score = 9–10), good (score = 11–12), very good (score = 13–14), and excellent (score = 15–16) [27]. In cases where the reviewers did not agree on the differences in scores, a third reviewer acted as an arbiter.

### Outcome measures

For APA onset measurements, we required studies to report the onset of muscle activity or onset COP displacement, relative to the beginning of the disturbance. For APA amplitude measurements, studies had to contain information on muscle recruitment within the anticipatory period. Muscle activity or COP onset or latency is defined in the literature as the beginning of the activity in relation to the beginning of the perturbation. Usually, it is set in terms of ±2 *SD* from the baseline activity (during quiet stand), visual inspection, or both. APA amplitude (or integral) is defined as the magnitude of the muscular activity, or COP displacement in 150 ms intervals, before the beginning of the perturbation, excluded the baseline activity.

Outcome measures we deemed to be acceptable for these purposes were as follows:

- Amplitude and duration (onset) of the postural muscles’ activity
- Amplitude and duration (onset) of the COP in the APA period
- Kinematic amplitudes included the displacement, frequency, duration, speed or acceleration of the arm, pendulum, or mobile platform

Acceptable methods for determining the onset of EMG, COP, or kinematics included methods based on computational analysis or visual identification. In cases where the data for meta-analysis were not entirely in the original article, we contacted the authors or obtained the data indirectly through the article’s published graphics.

### Data collection

Two reviewers extracted, independently, from each article data pertaining to participant characteristics, including sample size, experimental group configurations, type of balance disturbance, posture during the disturbance, different conditions adopted, means of recording, and statistical analyses methods. References collection and management were performed using Mendeley v1.19.6 (Mendeley Ltd., Elsevier, the Netherlands), and Microsoft Excel was used for screening and data extraction.

### Strategy for data synthesis

To analyze the aggregated results from these studies, we performed the analysis based on the outcome measures (EMG, COP, or kinematic measures) used in individual studies. The size of the intervention effect (Z) measure between younger and older adults was calculated for each study included in this review using mean difference (MD), since the intervention (perturbation protocol) in all studies is on the same direction. We calculated the value effect measure between younger and older adults for mean differences (and 95% confidence intervals), with statistical method inverse variance, and an analysis model carried out according to the heterogeneity. The heterogeneity of the included literature was tested using chi-square (*χ*^2^) test. It is statistically significant as *p* < 0.1. I squared (*I*^2^) statistics were used to quantify heterogeneity among the studies. When *I*^2^ < is 50%, it will be considered that there was no significant heterogeneity between studies, and the fixed effects model can be used [28]. We interpreted estimated effect sizes according to Cohen (1988; small ≤ 0.2, medium = 0.5, and large ≥ 0.8). Egger’s test was conducted to evaluate whether this meta-analysis had publication bias. If *p* < 0.05, it represented that there might exist publication bias. Two-tailed *p*-values were used in this study. Statistical analyses were performed using Review Manager (RevMan) 5.4.1 and Jamovi Version 2.2.5 software.

## Results

### Articles selected

**Fig. 1:**
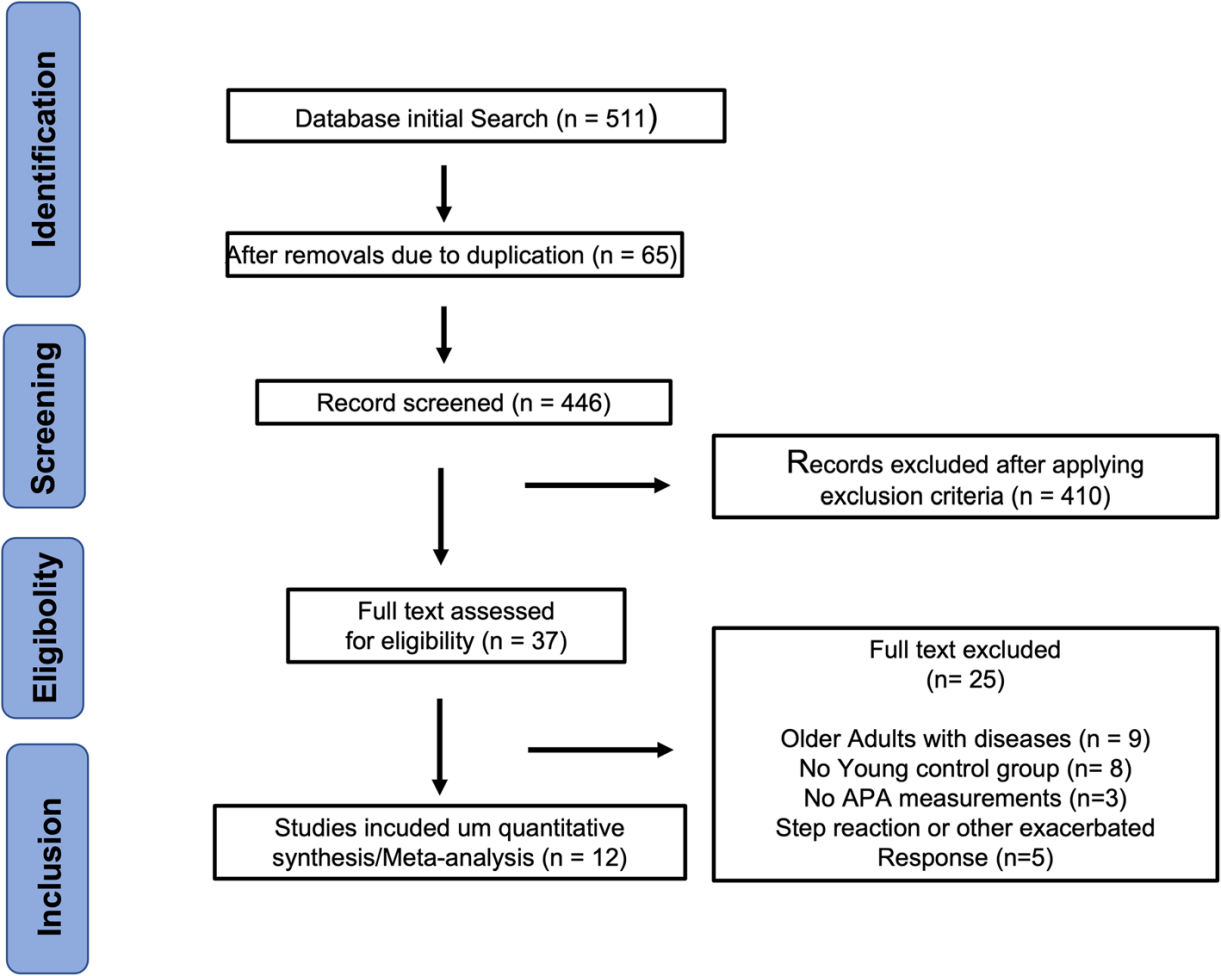
PRISMA flow diagram illustrating initial bibliographic research for article selection

Our initial systematic search via the named databases identified 511 articles. Applying exclusion criteria to the title and abstract and eliminating duplicate articles and articles that did not present a comparison between younger and healthy older adults and/or did not investigate APAs left us with 36 papers that were eligible for full reading. Data that remained non-recoverable (i.e., when authors did not return contact) were not entered into this meta-analysis (latencies of tibialis anterior (TA) muscles [4 studies], soleus (SOL) [1 study], gastrocnemius (GAS) [1 study], biceps femoris (BF) [1 study], erector spinae (ES) [1 study], and COP onset [1 study]). Reviewers demonstrated more than 90% agreement for which articles to include. A summary of these search results is presented in Fig. 1.

### Participants, objectives, and studied movements

The total number of participants across these 11 studies was 324, including 142 younger adults and 182 older adults. Table 2 summarizes the characteristics of the research protocols, posture during the perturbation, experimental conditions, outcome measures, recording methods, and the muscles or movements analyzed.

**Table 2:** Characteristics of the protocols, posture during the perturbation, conditions, outcome measures, recording methods, and the muscles analyzed

Study	Group	N (M/F)	Perturbation	Posture	Conditions	Reported outcomes
(a1) Kubicki et al., 2016 [29]	Mild cognitive impairment	14 (6/8)	Unilateral arm movement (pointing a led)	Standing	Simple/self-paced Predictable/unpredictable	Kinematic (1) Maximal velocity of the index movement (m/s) EMG (2) Muscles onset (RF, BF, OI, and ES) with reference to activation of the DA (3) % trials with APA (percentage for each participant)
	YA	14 (9/5)				
	OA	14 (5/9)				
(a2) Huang and Brown, 2013 [21]	YA	14	Unilateral arm movement (pick up and grasp a cylinder)	Standing	Simple/Self-paced Complex task Predictable	Kinematic (1) Movement speed (m/s) Force platform* (2) COP onset (s) (3) COP amplitude (cm) Other outcomes (4) Jerk score
	OA	16				
(a3) Woollacott and Manchester, 1993 [13]	YA	16 (8/8)	Unilateral arm movement (pointing a led)	Standing	Reaction time/complex task Predictable	Kinematic (1) Movement speed mean (m/s) EMG (2) Muscle onset (HAM, ES, Q, DA)
	OA	16 (8/8)				
(a4) Bleuse et al., 2005 [30]	YA	10 (5/5)	Unilateral arm movement (reach and grasp a handle)	Standing	Simple/self-paced Reaction time/complex task Predictable	Kinematic (1) Velocity peak (deg s) (2) Time of peak (s) (3) Movement duration (s) Force platform (4) COP onset (s) (5) COP amplitude (mm) (6) Time of the maximal displacement COP (s) (7) Onset (s) vertical torque (8) Peak vertical torque (N mm) (9) Area prior t0 (N mm s) (10) Area (N mm s) (11) Duration vertical torque (s) EMG (12) Muscle onset (BF, Q, TA, SOL, DA) (13) % of subjects activating a given muscle
	OA	10 (8/2)				
(a5) Lee et al., 2015 [18]	YA	8 (5/3)	Trunk perturbation (push a pendulum forward, using only trunk motion)	Standing	Simple task/self-paced Predictable	Force platform (1) COP onset (s) (2) COP amplitude (mm) (3) COP CPA: the COP peak values (mm) EMG (4) Muscle onset (TA, GAS, RF, BF, RA, ES) (5) APA integral of baseline activity Other outcomes (6) C indexes describe co-activation (7) R indexes describe reciprocal activation of agonist–antagonist muscle pairs
	OA	8 (4/4)				
(a6) Kanekar and Aruin, 2014 [15]	YA	13 (7/6)	Trunk perturbation (pendulum anterior impact)	Standing	Simple task/self-paced Predictable	Force platform* (1) COP amplitude (mm) (2) COP CPA: the COP peak values (mm) (3) Time of the maximal displacement COP (s) EMG* (4) Muscle onset (SOL, GAS, TA, RF, VM, VL, BF, ST, GL max, EO, RA, and ES) (5) APA integral of baseline activity Other outcomes (6) COM at tzero (7) COM peak (8) Time to COM peak
	OA	10 (6/4)				
(a7) Claudino et al., 2013 [19]	YA	20	Trunk perturbation (pendulum lateral impact)	Standing	Simple task/self-paced Predictable/unpredictable	Force platform (1) COP amplitude mediolateral (mm) (2) COP amplitude anteroposterior (mm) EMG (3) APA integral of baseline activity (RF, BF, GL Med, OI, RA, and ES)
	OA fallers	20				
	OA	11 (6/5)				
(a8) Inglin and Woollacott, 1988 [11]	YA	15 (8/7)	Unilateral arm movement (pull or push a manipulandum)	Standing	Simple task/self-paced Reaction time/complex task Predictable/unpredictable	EMG (1) Muscle onset (GAS, TA, HAM, Q, and RA)
	OA	15 (8/7)				
(a9) Laessoe and Voigt, 2007 [7]	YA	14	Tilt protocol: displacement in inclination Slide protocol: horizontal slide of the platform in the sagittal plane	Standing	Simple task/self-paced Reaction time/complex task Predictable	Force platform (1) COP amplitude (mm): passive body inertia related to the platform movement (2) COP CPA: the COP peak values (mm) Other outcomes (3) Frequencies of step reactions (%)
	OA	10				
(a10) Bugnariu and Sveistrup, 2006 [20]	YA	8 (4/4)	Slide protocol: horizontal slide of the platform in the sagittal plane	Standing	Reaction time/complex task Predictable/unpredictable	EMG* (1) Muscle onset (TA, GAS, Q, HAM, and ES) Other outcomes (4) Steps: the total number of steps (exacerbated response) (5) COP percentage: percentage of time the COP resided in one particular region reported
	OA	8 (4/4)				
(a11) Aloraini 2019 [31]	YA	10 (7/3)	Pointing task	Standing	Reaction time/complex task Predictable	Kinematic (1) Movement time (s) (2) Peak velocity (mm/s) (3) Time to peak velocity (ms) EMG (4) Muscle onset (TA and SOL) (5) APA amplitude
	OA	10 (7/3)				

*We contacted the authors or obtained the data indirectly through the article’s published graphics. *YA*, young adults; *OA*, older adults; *COM*, center of mass; *COP*, center or pressure. *TA*, tibialis anterior; *SOL*, soleus; *GAS*, gastrocnemius; *BF*, biceps femoris; *Q*, quadriceps; *RA*, rectus abdominis; and *Es*, erector spinae. *RF*, rectus femoris; *DA*, deltoid anterior; *OI*, obliquos internus; *VM*, vastus medialis; *VL*, vastus lateralia; *GL max*, gluteus maximus; *GL med*, gluteus medius; and *HAM*, hamstrings

In these studies, all paradigms used to generate APAs were performed with participants in an orthostatic position, but studies employed such different movements or disturbance mechanisms as follows: unilateral arm movement [11, 12, 14, 21, 32], pendulum impact or trunk flexion to push the pendulum [15, 18], basis of support oscillation using force platform [7, 20], and pointing task with the foot [31]. The experimental conditions involved reaction time or self-paced paradigms in self-initiated perturbations (i.e., to perform a task from a visual or auditory command, using maximum speed), simple or complex conditions (i.e., with additional challenges as double task, load increasing, or targets with different width), and predictable or unpredictable conditions (i.e., with or without vision and externally or self-triggered perturbations).

The studies’ outcome measurements included variables extracted from sEMG records [11, 12, 14, 15, 18–20, 31, 32], COP variables measured from a force platform [7, 14, 15, 18–21] or kinematic features [7, 12–15, 18–21, 31].

### Assessment of methodological quality

The scores obtained by applying the McMaster critical review form ranged from 5 to 13 out of a maximum possible score of 16. The Kappa agreement index between the evaluators was 0.825. This process resulted in one study being classified as poor, five rated as good quality, and five classified as very good (see Table 3). Named strengths of these selected studies included a clear methodology, adequate justification, good outline of the results, and an appropriate conclusion. Study weak points were muscles for which the location of the electrodes was not clearly discriminated, use of divergent methods across studies to define the APA period (i.e., establishing the beginning of the perturbation), and tendencies to draw conclusions using invalid methods.

**Table 3:** Methodological assessment using the McMaster Critical Review Form for Quantitative Studies

Author (year)	A	B	C	D	E	F	G	H	I	J	K	L	M	N	O	P	Scores	Rating	*NHMRC Level of evidence
Kubicki et al., 2016 [29]	1	1	1	1	1	0	1	1	1	1	0	1	1	1	0	1	13	Very good	III
Huang and Brown, 2013 [21]	1	1	1	1	1	0	1	1	1	1	0	1	1	1	0	1	13	Very good	III
Woollacott and Manchester, 1993 [13]	1	1	1	1	1	0	1	1	1	0	0	1	1	1	0	1	12	Good	III
Bleuse et al., 2005 [30]	1	1	1	1	1	0	1	1	1	0	0	1	1	1	0	1	12	Good	III
Lee et al., 2015 [18]	1	1	1	1	1	0	1	1	1	1	0	1	1	1	0	1	13	Very Good	III
Kanekar and Aruin, 2014 [15]	1	1	1	1	1	0	1	1	1	1	0	1	1	1	0	1	13	Very Good	III
Claudino et al., 2013 [19]	1	1	1	1	1	1	1	1	1	0	0	1	1	1	0	1	13	Very Good	III
Inglin and Woollacott, 1988 [11]	1	1	1	1	0	0	0	0	0	0	0	1	0	0	0	0	5	Poor	III
Laessoe and Voigt, 2007 [7]	1	1	1	1	1	0	1	1	1	1	0	1	1	0	0	1	12	Good	III
Bugnariu and Sveistrup, 2006 [20]	1	1	1	1	1	0	1	1	1	0	0	1	1	1	0	1	12	Good	III
Aloiraini, 2019 [31]	1	1	1	1	1	1	1	1	1	0	0	1	1	1	0	1	13	Very Good	III

1 criteria fulfilled completely, 0 criteria not fulfilled completely. Quality category: poor (≤ 8), fair (9–10), good (11–12), very good (13–14), and excellent (15–16). *A level of evidence as per the hierarchy of evidence. The McMaster Critical Review Form for Quantitative Studies [26]. Citation: provided the full citation for this article in APA format? (A). Study purpose: was the purpose and/or research question stated clearly? (B). Literature: was relevant background literature reviewed? (C). Study design: was a theoretical perspective identified? (D). Sampling: was the process of purposeful selection described? (E). Was sampling size justified? (F). Outcomes: were the outcome measures reliable? (G). Outcomes: were the outcome measures valid? (H). Intervention: intervention was described in detail? (I). Intervention: contamination was avoided? (J). Intervention: cointervention was avoided? (K). Results: results were reported in terms of statistical significance? (L). Results: were the analysis method(s) appropriate? (M). Results: clinical importance was reported? (N). Results: dropouts were reported? (O). Conclusions and implications: conclusions were appropriate given study methods and results (P)

### Muscle activity onset (latency)

**Fig. 2:**
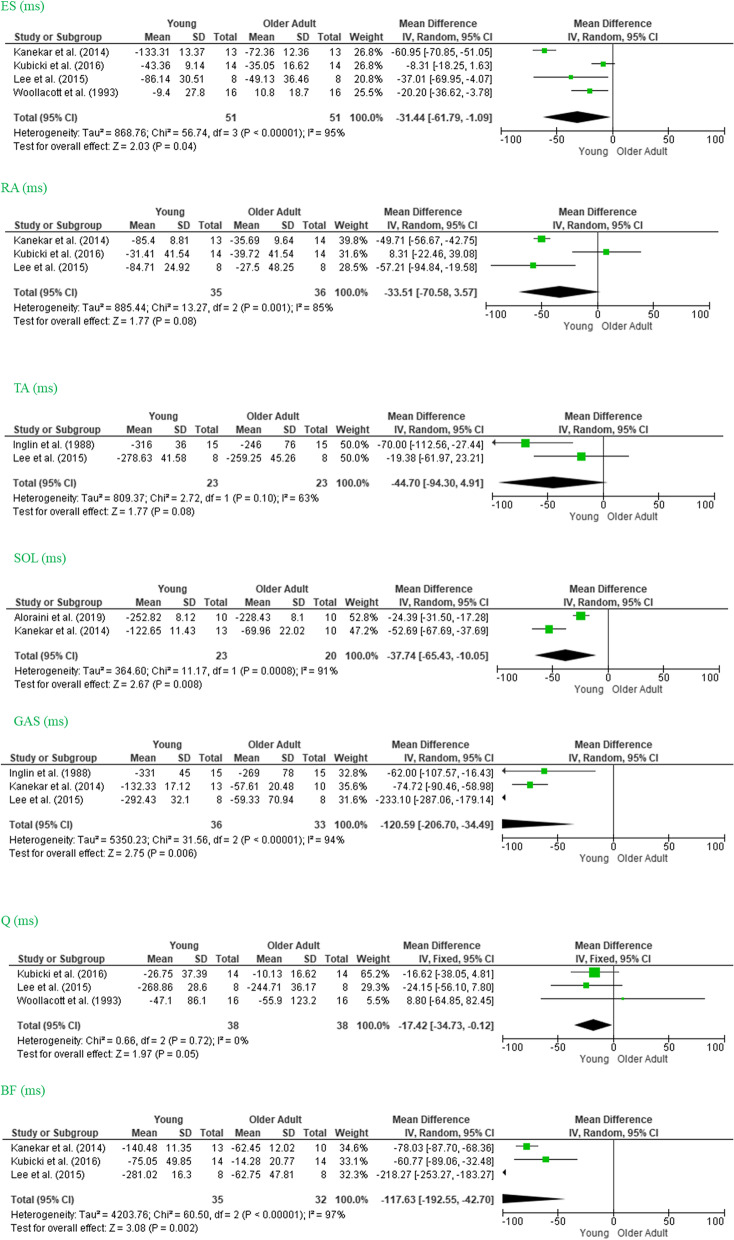
Forest plot of muscles onset. Note: MD, mean difference. T, tibialis anterior; SOL, soleus; GAS, gastrocnemius; BF, biceps femoris; Q, quadriceps; RA, rectus abdominis; and Es, erector spinae

Muscle activity onset, defined in the literature as the beginning of muscle activity in relation to the beginning of the perturbation, was the most frequently used variable across studies (reported in nine studies). Authors measured this variable in terms of ±2 *SD* from the baseline activity (during quiet stand), visual inspection, or both. However, some authors did not assess the beginning of the perturbation precisely as a reference for APA calculations, and this omission compromised our quantitative synthesis. The final latency of lower leg, thigh, and trunk muscles is shown in Fig. 2. The SOL, GAS, Q, BF, and ES muscles had a significant effect size, with more anticipation evident among younger than among older participants. This analysis showed that the Q did not present significant heterogeneity: Q (*MD* −17.42, 95% *CI* −34.73, −0.12, *I*^2^ = 0%). However, ES, BF, RA, TA, SOL, and GAS showed high heterogeneity with a high extent of dispersion in the size of the true effect across studies: ES (*MD* −31.44, 95% *CI* −61.79, −1.09, *I*^2^ = 95%); BF (*MD* −117.47, 95% *CI* −192.55, −42.70, *I*^2^ = 97%); RA (*MD* −31.51, 95% *CI* −70.58, −3.57, I2 = 85%); TA (*MD* −44.70, 95% *CI* −94.30, 4.91, *I*^2^ = 63%); SOL (*MD* −37.74, 95% *CI* −65.43, −10.05, *I*^2^ = 91%); GAS (*MD* −120.59, 95% *CI* −206.70, −34.49, *I*^2^ = 94%).

### Muscle activity amplitude (APA integral)

The electromyographic integral (iEMG) measurement was an infrequently used parameter in these studies, and, isolated, it was not efficient as a means of demonstrating differences between groups in APA periods. The iEMG in APA measurements is defined as the magnitude of the muscular activity in 150 ms intervals, before the beginning of the perturbation, excluded the baseline activity. Four articles measured this variable, but the great variation between the muscles studied and differences in the measurement interval or in the way of reporting the result made it impossible to perform a meta-analysis on this variable. The values of this variable are reported in Table 4.

**Table 4:** iEMG measurements in APA epochs

Study	Outcome	MD (± 95% *CI*)
Aloraini et al. (2019) [31]	SOL	2.32 (2.06, 2.58)
Kanekar et al. (2014) [15]	GAS	0.48 (0.38, 0.58)
	ST	0.36 (0.20, 0.52)
	ES	0.40 (0.30, 0.50)
Claudino et al. (2013) [19]	OE	0.03 (0.01, 0.05)
	GLUTEO MED	0.01 [−0.00, 0.02)
Lee et al. (2015) [18]	C-Rindex	−0.42 (−3.70, 2.86)
	Rindex	0.00 (−3.28, 3.28)

### COP onset and amplitude

**Fig. 3:**
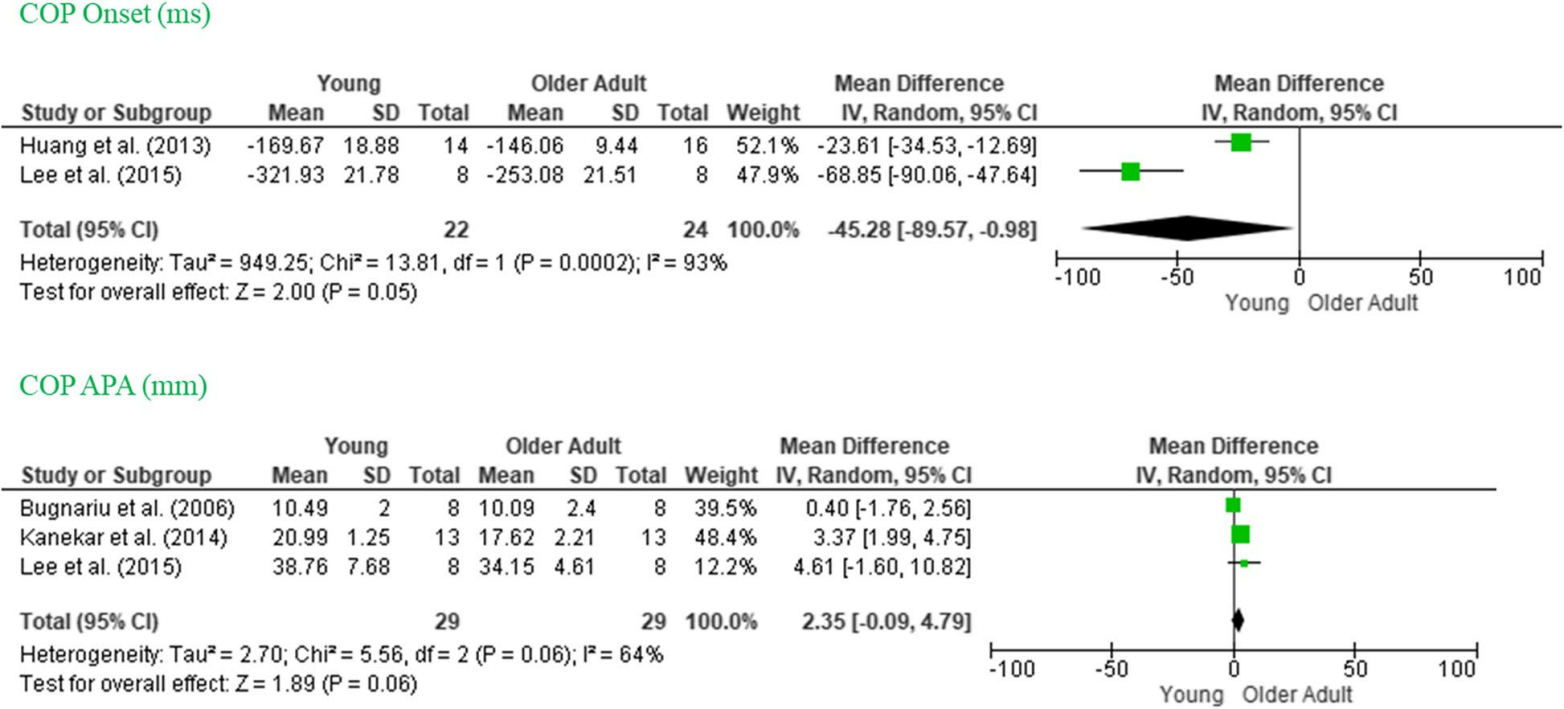
Forest plot of COP onset and COP amplitude (COP APA) for selected studies. Note: MD, mean difference

COP onset is defined as the beginning of the displacement of the COP in relation to the beginning of the perturbation. There was a significant effect size for COP onset (*p* = 0.002), and this movement was anticipated more in the younger adult than in the older adult group. It did not show significant heterogeneity COP onset (*MD* −45.28, 95% *CI* −89.57, −0.98, *I*^2^ = 93%). The COP amplitude is the magnitude of the posterior displacement, measured from the onset to the beginning of the perturbation. For this variable, there was no significant effect size (*p* = 0.06): COPapa (*MD* 2.35, 95% *CI* −0.09, 4.79, *I*^2^ = 64%). The values of these COP variables are shown in Fig. 3.

### Kinematic measurements

**Fig. 4:**
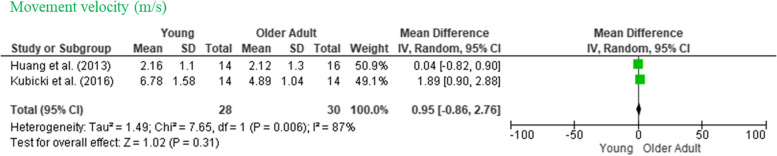
Forest plot of the movement velocity of participants across studies. Note: M, mean difference

Kinematic measures were reported in 10 of these 11 articles, essentially to set the moment when the perturbation started. Three studies reported the maximum movement speed during the task. One of them was measured in degrees/second with no difference between groups’ MD (± 95% *CI*): 0.30 [−0.39, 1.00] [13]. There was no significant difference between the groups (*MD* 0.95, 95% *CI* −0.86, 2.76, *I*^2^ = 82%), as can be seen in Fig. 4.

### Testing for publication bias

Egger’s test (*p* < 0.05) was used to detect the publication bias in analyses with more than two studies. We observed that the analysis was not asymmetric for Q (ms) (Egger’s regression = −1.105, *p* = 0.269) and COP APA (mm) (Egger’s regression = −1.552, *p* = 0.121), suggesting that publication bias might exist for the other analyses: ES (ms) (Egger’s regression = −3.467, *p* < 0.0001); RA (ms) (Egger’s regression = −6.061, *p* < 0.0001); GAS (ms) (Egger’s regression = −4.338, *p* < 0.0001); BF (ms) (Egger’s regression = −5.163, *p* < 0.0001).

## Discussion

The aim of the present literature review of 11 research studies selected for their relevance and methodological quality was to examine the effect of age on postural muscle control based on the measurement of APAs involving expected anteroposterior perturbations. Our overall analysis of muscles and COP onset times showed that anticipatory postural control was more evident among younger adult participants in past studies, compared to older adult participants. This suggests that older adults may have altered postural control mechanisms associated with age. This evidence was limited, however, due to the variability of methods employed for data processing in these studies and the fact that some investigators used omitted certain calculations or definitions of parameters, meaning that these studies could not be included in the meta-analysis. In addition, kinematic evidence to suggest altered APAs among older adults remains inconclusive, largely due to the scarcity of available studies. Latency, or muscle activation onset, is the time measured between a stimulus and the effective start of a response, which allows the investigation of any change from the baseline [33, 34]. In this review, we observed that muscle onset was the most frequently used measurement method in the investigation of APAs. In addition, for trunk, leg, and thigh muscles, there was evidence of more anticipation among younger than among older participants. Mainly, there was a significant difference found in SOL, GAS, Q, BF, and ES muscles. These results support an aging process that contributes to a consequent decline in balance systems that influence APAs. By studying the onset of the postural muscles, investigators have been able to assess muscle synergy in postural control, necessary to perform movement. Few studies, however, have discussed the differences in the order of the recruitment or the patterns of muscle recruitment between younger and older participants [11, 13, 14, 18, 29, 35]. In general, studies have reported that these muscles have a similar behavior (i.e., distal to proximal and reciprocal activation distal) in younger adults with significant anticipation, compared to older adults [11, 13, 14]. While one study showed that young people have a pattern of distal to proximal muscle recruitment (i.e., hamstrings, spinal erector, and anterior deltoid), older adults demonstrated a different but nonspecific order, sometimes showing initiation by proximal muscles and with a co-contraction pattern [11, 13, 14, 18].

In addition to muscle activation onset, researchers have sometimes used iEMG to quantify the amount of muscle activation, relying on an assessment of the area on the signal rectified curve [36]. In these studies, there was no significant difference in the muscle amplitude during APA period. However, in comparing older and younger participants, there was an increase in muscle magnitude among the older adults compared to younger adults in the compensatory period (i.e., after the disturbance) [15, 19]. One of the ways used to calculate iEMG [18] is to employ the co-activation and the reciprocal activation indexes (C and R, respectively), where the C index represents co-activation and the R index represents the reciprocal activation of agonist and antagonist muscle groups [37, 38]. In our collective analysis of these studies, during the APA phase, younger adults used less co-activation strategy and more reciprocal activation (lower C index than R index) than did older adults for whom there was no difference in the strategy found. During the compensatory phase, on the other hand, co-activation was mainly employed by older adults in the leg, thigh, and trunk segment muscles, while the younger adults continued to use reciprocal activation. The absence of inhibition in older adults means that their central nervous system employed the co-contraction strategy due to the high demanding task [39] that caused a more challenging perturbation for them, compared to younger adults.

Postural control in the older adults can undergo changes because of neuromotor and sensorial changes that occur with the aging process. These modifications can cause difficulty in receiving and processing sensorial information, as well as in the execution and motor control during upright posture [40]. The aging process can lead to changes in the peripheral nervous system, with less plantar afferences [41] and proprioceptive inputs [42] for the somatosensory system that undergoes difficulties both to detect movements and changes in muscle length and tension [43–45]. In addition, there is a decrease in the ability to generate explosive contractions, due to a decrease in type 2 fibers [46]. With the decrease in the number and size of type 2 muscle fibers, there is a reduction in the production of quick muscle strength during reactive movements [47] and during rapid postural adjustments [40]. Another factor related to the decrease in the generation of muscle strength corresponds to neuronal factors associated with decreased numbers of motor neurons and the ability to send impulses and activate the motor units [46, 47]. Therefore, aging can alter the organization of onset and the muscle magnitude of anticipatory postural control [15]. Delayed or decreased APA responses can influence the performance of motor activities in older adults. Some authors, for example, demonstrated longer reaction time during the execution of movements after a go signal, compared to younger adults [11, 32, 33].

The use of force platform to investigate the COP displacement is also a common method described in this review. The COP is the displacement influenced by the center of mass [48], which corresponds to the point where the resulting vertical force is applied on the support surface [49]. This review showed that younger participants in past research had a higher COP onset compared to older adult participants with a heterogeneity index of *I*^2^ 61%, and this shows a delay in the beginning of COP displacement in the older adults group. Forward-oriented movements are known to be associated with a backward displacement of the COP that starts before the movement and is related to the speed of the movement or amount of disturbance [30, 50, 51]. Regardless of the paradigm in which the authors of past studies provided participants with substantial imbalance, the displacement of COP for the older adult participants did not occur or occurred late, leading to postural disorganization, with losses in the execution of the movement [18]. The amount of the COP displacement during the APA period (i.e., 150 ms before the beginning of the perturbation) is known as COP APA [18]. Few authors have investigated this measurement, and there is no consensus finding in the literature. Some investigators reported that younger participants had greater amplitude in this period, resulting in less displacement in the compensatory period [52]. Our meta-analysis showed no significant effects when comparing healthy younger and older adults. Older adults showed similar displacement of the COP in the APA period than younger adults, and this did not result in a better balance recovery at the compensatory moment. So, the inability to control the magnitude and displacement of the COP combined with the delay in muscle onset and COP can predispose older adults to greater instability, making it more difficult for them maintain balance after a perturbation [18].

Healthy older adults can produce APAs in the face of predictable perturbations, but this mechanism does not have the expected effect of decreasing compensatory activity, as compensation is less effective in older adults than in younger adults [19]. Huang and Brown [21] found that even when the COP APA was higher among older adults, this finding did not result in less displacement in the compensatory phase. The lower effectiveness of APAs in the older adults may be related to structural and biochemical changes in areas of the neural system and in structures important for APAs to be generated, such as a supplementary motor area and the foot representation area in the sensorimotor cortex, causing greater instability of posture among older adults [15]. In addition, tasks with cognitive demand, without clues to the beginning of the disturbance, fear of falling, and focus of internal attention, can all restrict automatic control processes, which are faster, and this restriction also interferes with movement adjustments [7, 19, 20, 31].

Finally, the kinematic results in this review indicate that older adults in past research were able to perform movements with a magnitude of perturbation comparable to younger adults, demonstrating that the differences observed in muscle activity and COP displacements between groups can be attributed to age effects [13, 14, 18, 21]. While this decline in age-related motor control might be expected to lead to lower task, older adults may use alternative strategies to perform movements similarly to younger adults [14]. To perform the movement according to the requirements imposed, the older adults make use of different strategies that aim to increase the safety margin and stabilize the body to perform the movement [21]. Older adults can alter the sequence of muscle activation, differing from young people who commonly started from distal to proximal [14]. Older adults used a co-contraction strategy instead of reciprocal activation [18] and made greater adjustments during movement, leading to greater alternation between acceleration and deceleration than younger adults [31].

Finally, we recommend caution when interpreting all these results requires caution. As presented, there are few studies in the literature comparing health of young and older subjects, with a comparable design. We just included for meta-analyses anteroposterior perturbation, with kinematic measurements for setting the tzero. Even so, the variations in the movements (pointing, grasping, pendulum impact) and disturbance mechanisms employed in the included studies may be responsible in part by the significant heterogeneity found in some outcomes. Any generalization should be avoided.

### Limitations and directions for further research

Among potential limitations of this study was that few selected studies performed statistical power analyses to calculate required sample sizes. Adjusted estimates are necessary in non-randomized studies to adjust for confounding; however, in the present paper, no adjusted estimate was performed due to the number of students included in this meta-analysis. Our meta-analysis then risks bias and can lead to errors. Thus, it is necessary to exercise considerable caution when interpreting it. The use of different variables and parameters in the studies reviewed made a meta-analysis with more robust values infeasible. The main metrics for assessing APA were included in this review; however, research has shown that there is no standard or most valid way to assess APA. Researchers should be aware that most studies investigating APA use some of the variables analyzed in this study; however, it is not clear whether these results are generalizable for different experimental models, postural conditions, assessed task, or associated pathologies. Finally, our selection narrowed the literature on this topic to only 11 relevant and well-conducted studies, meaning that this is still a new science, with results that may change as further research ensues.

## Conclusion

The changes in motor responses found in the face of an expected perturbation in past research do not seem to be linked to the speed needed to perform a movement but may be linked to physiological changes that occur in senescence in that older adults have shown decreased motor control during APAs. Our review and meta-analysis found that muscle and COP onsets in response to expected perturbations were delayed in older compared to young adult research participants, suggesting that APAs are altered in the older adult population. However, the standardization of variables and measurements used to assess APA needs to be carefully verified by authors in future studies, and we discuss several important limitations to this review.

## Supplementary Information

**Additional file 1: Supplementary Table S1.** Prism Checklist.

**Additional file 2: Supplementary Table S2.** Search Strategy.
