# Supplementary material for: Anticipatory postural adjustments in older versus young adults: a systematic review and meta-analysis
Source: Syst Rev. 2022 Nov 23;11:251. doi: 10.1186/s13643-022-02116-x (PMC9685888; doi:10.1186/s13643-022-02116-x)
Supplement: Supplementary file 2 — Additional file 2: Supplementary Table S2. Search Strategy. [file 13643_2022_2116_MOESM2_ESM.docx]

**Supplementary Table S2. Search Strategy for Databases.**

| **Database** | **Search strategies** | **Results** |
| --- | --- | --- |
| Pubmed | (APAs) AND (aging) "APAs"[All Fields] AND ("aging"[MeSH Terms] OR "aging"[All Fields] OR "ageing"[All Fields]) | 34 |
|  | (anticipatory postural adjustments) AND (aging)"anticipatory"[All Fields] AND ("postural"[All Fields] OR "posturally"[All Fields] OR "posture"[MeSH Terms] OR "posture"[All Fields] OR "postures"[All Fields] OR "postured"[All Fields] OR "posturing"[All Fields]) AND ("adaptation, psychological"[MeSH Terms] OR ("adaptation"[All Fields] AND "psychological"[All Fields]) OR "psychological adaptation"[All Fields] OR "adjustment"[All Fields] OR "adjust"[All Fields] OR "adjusted"[All Fields] OR "adjusting"[All Fields] OR "adjustments"[All Fields] OR "adjusts"[All Fields]) AND ("aging"[MeSH Terms] OR "aging"[All Fields] OR "ageing"[All Fields]) | 79 |
|  | (APAs) AND (elderly)"APAs"[All Fields] AND ("aged"[MeSH Terms] OR "aged"[All Fields] OR "elderly"[All Fields] OR "elderlies"[All Fields] OR "elderly s"[All Fields] OR "elderlys"[All Fields]) | 331 |
|  | (anticipatory postural adjustments) AND (elderly)"anticipatory"[All Fields] AND ("postural"[All Fields] OR "posturally"[All Fields] OR "posture"[MeSH Terms] OR "posture"[All Fields] OR "postures"[All Fields] OR "postured"[All Fields] OR "posturing"[All Fields]) AND ("adaptation, psychological"[MeSH Terms] OR ("adaptation"[All Fields] AND "psychological"[All Fields]) OR "psychological adaptation"[All Fields] OR "adjustment"[All Fields] OR "adjust"[All Fields] OR "adjusted"[All Fields] OR "adjusting"[All Fields] OR "adjustments"[All Fields] OR "adjusts"[All Fields]) AND ("aged"[MeSH Terms] OR "aged"[All Fields] OR "elderly"[All Fields] OR "elderlies"[All Fields] OR "elderly s"[All Fields] OR "elderlys"[All Fields]) | 301 |
|  | (APAs) AND (older)"APAs"[All Fields] AND ("older"[All Fields] OR "olders"[All Fields]) | 40 |
|  | (anticipatory postural adjustments) AND (older)"anticipatory"[All Fields] AND ("postural"[All Fields] OR "posturally"[All Fields] OR "posture"[MeSH Terms] OR "posture"[All Fields] OR "postures"[All Fields] OR "postured"[All Fields] OR "posturing"[All Fields]) AND a("adaptation, psychological"[MeSH Terms] OR ("adaptation"[All Fields] AND "psychological"[All Fields]) OR "psychological adaptation"[All Fields] OR "adjustment"[All Fields] OR "adjust"[All Fields] OR "adjusted"[All Fields] OR "adjusting"[All Fields] OR "adjustments"[All Fields] OR "adjusts"[All Fields]) AND ("older"[All Fields] OR "olders"[All Fields]) | 80 |
|  | (APAs) AND (old)"APAs"[All Fields] AND "old"[All Fields] | 32 |
|  | (anticipatory postural adjustments) AND (old)"anticipatory"[All Fields] AND ("postural"[All Fields] OR "posturally"[All Fields] OR "posture"[MeSH Terms] OR "posture"[All Fields] OR "postures"[All Fields] OR "postured"[All Fields] OR "posturing"[All Fields]) AND ("adaptation, psychological"[MeSH Terms] OR ("adaptation"[All Fields] AND "psychological"[All Fields]) OR "psychological adaptation"[All Fields] OR "adjustment"[All Fields] OR "adjust"[All Fields] OR "adjusted"[All Fields] OR "adjusting"[All Fields] OR "adjustments"[All Fields] OR "adjusts"[All Fields]) AND "old"[All Fields] | 21 |
|  | Peer Reviewed Published Date: 1900-2019 Narrowed by Language: -English |  |
|  |  |  |
| Lilacs via bvs | APAs [Palavras] and elderly [Palavras] | 1 |
|  | anticipatory postural adjustments [Palavras] and elderly [Palavras] | 0 |
|  | anticipatory postural adjustments [Palavras] and old [Palavras] | 0 |
|  | anticipatory postural adjustments [Palavras] and older[Palavras] | 0 |
|  | No filter |  |
|  |  |  |
| Scielo | Expressão: (APAs) AND (elderly)  Filtros aplicados: Published Date: 1900-2019 Narrowed by Language: -English | 0 |
|  | Expressão: (APAs) AND (older)  Filtros aplicados: Published Date: 1900-2019 Narrowed by Language: -English | 0 |
|  | Expressão: (anticipatory postural adjustments) AND (older)  Filtros aplicados: Published Date: 1900-2019 Narrowed by Language: -English | 0 |
|  | Expressão: (APAs) AND (elderly)  Filtros aplicados: Published Date: 1900-2019 Narrowed by Language: -English | 0 |
|  |  |  |
| Cochrane Central | anticipatory postural adjustments in Title Abstract Keyword AND older in Title Abstract Keyword - (Word variations have been searched) | 26 |
|  | anticipatory postural adjustments in Title Abstract Keyword AND elderly in Title Abstract Keyword - (Word variations have been searched) | 18 |
|  | APAs in Title Abstract Keyword AND elderly in Title Abstract Keyword - (Word variations have been searched) | 74 |
|  | APAs in Title Abstract Keyword AND older in Title Abstract Keyword - (Word variations have been searched) | 2 |
|  | Published Date: 1900-2019 Narrowed by Language: -English |  |
|  |  |  |
| Embase/Web of Science | (ALL=(anticipatory postural adjustments)) AND ALL=(old) | 201 |
|  | **(ALL=(APAs)) AND ALL=(elderly)** | 30 |
|  | (ALL=(APAs)) AND ALL=(old) | 84 |
|  | **(ALL=(anticipatory postural adjustments)) AND ALL=(elderly)** | 79 |
|  | Refined by: LANGUAGES: (ENGLISH) AND PUBLICATION YEARS: (1900 to 2019) |  |
